# Spray-Flame Synthesis (SFS) and Characterization of Li_1.3_Al_0.3−x_Y_x_Ti_1.7_(PO_4_)_3_ [LA(Y)TP] Solid Electrolytes

**DOI:** 10.3390/nano15010042

**Published:** 2024-12-29

**Authors:** Md Yusuf Ali, Hans Orthner, Hartmut Wiggers

**Affiliations:** 1 Institute for Energy and Materials Processes—Reactive Fluids, University of Duisburg-Essen, 47057 Duisburg, Germany; yusuf.ali@uni-due.de (M.Y.A.); hans.orthner@uni-due.de (H.O.); 2 Center for Nanointegration Duisburg-Essen (CENIDE), 47057 Duisburg, Germany

**Keywords:** solid-state electrolytes, lithium-ion batteries, spray-flame synthesis, nanoparticles, Lithium Aluminum Titanium Phosphate (LATP), ionic conductivity

## Abstract

Solid-state electrolytes for lithium-ion batteries, which enable a significant increase in storage capacity, are at the forefront of alternative energy storage systems due to their attractive properties such as wide electrochemical stability window, relatively superior contact stability against Li metal, inherently dendrite inhibition, and a wide range of temperature functionality. NASICON-type solid electrolytes are an exciting candidate within ceramic electrolytes due to their high ionic conductivity and low moisture sensitivity, making them a prime candidate for pure oxidic and hybrid ceramic-in-polymer composite electrolytes. Here, we report on producing pure and Y-doped Lithium Aluminum Titanium Phosphate (LATP) nanoparticles by spray-flame synthesis. The as-synthesized samples consist of an amorphous component and anatase-TiO_2_ crystalline particles. Brief annealing at 750–1000 °C for one hour was sufficient to achieve the desired phase while maintaining the material’s sub-micrometer scale. Rietveld analysis of X-Ray diffraction data demonstrated that the crystal volume increases with Y doping. At the same time, with high Y incorporation, a segregation of the YPO_4_ phase was observed in addition to the desired LATP phase. Another impurity phase, LiTiOPO_4_, was observed besides YPO_4_ and, with higher calcination temperature (1000 °C), the phase fraction for both impurities also increased. The ionic conductivity increased with Y incorporation from 0.1 mS/cm at room temperature in the undoped sample to 0.84 mS/cm in the case of LAY0.1TP, which makes these materials—especially considering the comparatively low sintering temperature—highly interesting for applications in the field of solid-state batteries.

## 1. Introduction

Lithium-ion batteries (LIBs) have become indispensable for powering modern electronic devices and autonomous systems due to their lightweight, high-energy density and superior power output. However, most commercial LIBs rely on liquid electrolytes and organic polymer separators, which introduce several limitations. These include poor oxidative stability, high flammability, and incompatibility between lithium metal anodes and the electrolyte [1]. Replacing liquid electrolytes with solid-state electrolytes has emerged as a promising solution to overcome these challenges [2]. Inorganic solid electrolytes offer high thermal stability, improved compatibility with lithium metal anodes, and high ionic conductivity. The solid-state electrolyte covers various materials, including organic polymer, inorganic (mainly oxides and sulfides), and composites [3,4,5], such as a mixture of polymer [poly(vinylidene fluoride] and ceramic [Li_4_Ti_5_O_12_] electrolytes [6]. Within the last couple of decades, multiple types of inorganic oxidic electrolytes such as perovskite-type (Li_3x_La_2/3−x_TiO_3_, LLTO), garnet type (Li_7_La_3_Zr_2_O_12_, LLZO), and NASICON type (Li_1+x_Y_x_Zr_2−x_(PO_4_)_3_, LYZP) (Li_1+x_Al_x_Ge_2−x_(PO_4_)_3_, LAGP) as well as sulfide type systems such as thiophosphates (Li_3_PS_4_-type, i.e., Li_10_GeP_2_S_12_, LGPS) and argyrodite (Li_6_PS_5_Cl)-type solid electrolytes have been studied [7]. In the field of oxides, the NASICON-type materials with rhombohedral R3c structure are of interest due to their 3D ion transport structure; and respective materials, i.e., LiTi_2_(PO_4_)_3_ (LTP), have been intensively studied for Li-ion conduction since the early 1990s [8]. Despite the presence of sufficient diffusion paths, the ionic conductivity of LTP is still inferior to liquid-based electrolytes, and additional advancements in ionic conductivity are required [9]. Doping is a popular strategy to modify the electrochemical properties of electrolytes to increase the concentrations of the charge-carrying defect species through aliovalent doping [10]. Tuning the ionic conductivity of LTP can be done by substituting Ti^4+^ with other aliovalent cations such as Ca^2+^, Al^3+^, and isovalent cation doping such as Hf^4+^ [11,12]. Moreover, similar ionic conductivity improvements could be achieved by replacing P in tetrahedral sites with pentavalent vanadium and niobium [13]. Among these substituents, Al^3+^ has proven (Li_1+x_Al_x_Ti_2−x_(PO_4_)_3_, LATP) to be one of the most effective dopant variations while adjusting the cell volume and simultaneously reducing the Li-O bond strength to optimize lithium-ion transport [14]. Moreover, Al forms a secondary aluminum-rich AlPO_4_ impurity phase, strengthening densification and successfully forming a composite to increase overall ion conductivity. However, further increases in Al content have negative consequences, such as significantly increased residual lithium on the cathode surface and reduced initial capacity due to impurity phase formation (such as LiAlO_2_ and Li_5_AlO_4_) [15]. In addition, LATP demonstrates higher grain boundary impedance, leading to poor overall performance [16]. To mediate this, Kothari et al. proposed the formation of a space-charge region at the grain boundary due to the presence of impurities, which increases as the doping (Sc^3+^, Y^3+^) concentration is increased [17]. The authors attributed the space charge formation to the increasing diffusion coefficient and mean square displacement (from electrochemical impedance measurement). Thus, doped NASICON-type LTP has drawn scientific interest as a desirable inorganic SSE for cutting-edge electrochemical energy storage systems.

In addition to the material properties, the processing route is essential for performance since it affects the structural properties and phase components, determining ionic conductivity. Large-scale manufacturing of LATP should be possible without the need for complex processes, expensive raw materials, and significantly less pollutant emission. Furthermore, nanoparticles have been demonstrated to have high mechanical abilities, surface area, and significant shortening of ionic conductive pathways [18,19]. The standard synthesis routes of any ceramic electrolyte are mainly solid-state reactions and sol-gel processes with subsequent annealing [20,21,22]. However, these processes are either energy–time demanding (solid-phase synthesis) or limited in quantity yield. Since the sol-gel synthesis of nanoparticles is typically a multistep batch process, unlike continuous gas-phase synthesis, its scalability and, thus, its production rate is limited. Thus, spray-flame synthesis (SFS) can be an alternative process to synthesize functional nanoparticles with typically mono-modal particle size distribution and high phase purity [23,24]. Different diagnostic methods and corresponding theoretical models achieved a significant improvement in the mechanism of spray combustion based on metal oxide precursor solutions in the last few decades [25,26]. SFS can synthesize complex, multicomponent particles while keeping the precursor–component ratio. Moreover, SFS is a contestant as a continuous, easily scalable, low-cost alternative synthesis method for solid-state electrolytes [24]. Additionally, like other methods, SFS can be manipulated through energy supplied, reaction temperature, solvent/solute properties, reactor pressure, etc. The primary goal of this report is to demonstrate SFS as an alternative, continuous, easily upscaling synthesis route for producing nanosized NASICON solid electrolytes.

Reports on the synthesis of LATP by SFS are very few and far between. Shin et al. have synthesized LATP@Li_2_O-2B_2_O_3_ core-shell (>1 μm) spherical micrometer-sized dense particles by nebulized-spray-pyrolysis [27] with the highest conductivity of 1.519 × 10^−4^ S cm^−1^. Badding et al. patented synthesizing LATP and LAGP by SFS [28]. However, no detailed characterization of the as-synthesized samples was presented. Here, we have investigated the gas-phase synthesis of LATP nanoparticles via SFS and the subsequent calcination step required to produce the intended rhombohedral NASICON phase. Starting from Li_1.3_Al_0.3_Ti_1.7_(PO_4_)_3_, aluminum was gradually substituted with varying concentrations of Y^3+^ [Li_1.3_Al_0.3−x_Y_x_Ti_1.7_(PO_4_)_3_, x = 0.01 − 0.15] to further enhance the ionic conductivity of LATP. The materials produced were analyzed for phase composition, thermal properties, surface characterization, and ionic conductivity. With this report, SFS could be standardized as an alternative synthesis technique for the mass production of high ionic conductive LATP material.

## 2. Materials and Methods

The SFS reactor used for synthesizing the nanoparticles, which consists of a com-bination of spray and pilot flame, has been discussed in our previous work [24]. A closed reactor was used to ease pressure regulation and particle collection on the ePTFE-membrane-coated polyester filter. The premixed CH_4_/O_2_ pilot flame was used as an energy and ignition source. A precursor solution is atomized and ignited in a 2-fluid nozzle, with a CH_4_/O_2_ gas mixture serving as the dispersion gas. A sheath flow of compressed air was provided for flame stability and as a carrier gas for the product. Downstream the reaction zone, compressed air was used to quench the reaction and decrease the reactor temperature. The pressure was kept slightly below ambient pressure for directed product accumulation.

Precursors were used as supplied: LiNO_3_ (LN; VWR, ≥99.0% purity; Leuven, Belgium), Y(NO_3_)_3_∙6H_2_O (YN; Aldrich, ≥99.8% purity; Darmstadt, Germany), Titanium (IV) isopropoxide (TTIP) (Sigma-Aldrich, 97% purity; Mumbai, India) was used as the Ti source, Al(NO_3_)_3_ × 9 H_2_O, (AN; Merck; Leuven, Belgium) as Al source and Tributyl phosphate ((C_4_H_9_)_3_PO, TBP, Sigma Aldrich, ≥99% purity; Darmstadt, Germany) as phosphate source. Solvent mixtures were prepared from 2-propanol (PrOH, BASF, ≥99.5% purity; Ludwigshafen, Germany) and propionic acid (PA, Acros Organics, ≥99% purity; Geel, Belgium).

The precursor concentration was maintained at 0.5 M, and the precursor feeding rate to the reactor was 2 mL/min. To compensate for the Li loss during the calcination steps, 25 wt% excess Li was used [29]. The operating parameters are shown in Table 1, and the detailed reaction scheme for producing differently doped LATP is provided in Table 2.

**Figure 1:**
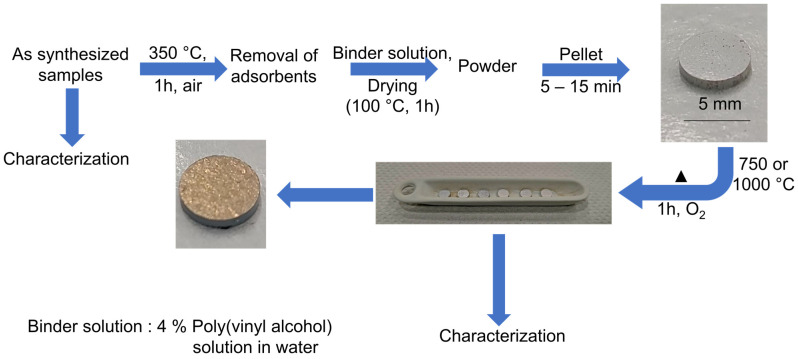
Scheme of sintered bodies preparation for the investigation of ionic conductivity.

The sample preparation steps for characterization are shown in Figure 1. Due to their high specific surface area, SFS synthesized samples generally have an unavoidable deposition of unburnt carbonaceous species and water adsorbents, which will be discussed later based on simultaneous thermal analysis (STA) results. Therefore, the as-synthesized samples are heated at ~350 °C under air for 1 h to remove these impurities. To process these materials into mechanically stable solid electrolyte pellets, the pre-heated samples were mixed with binder solution (4% PVA solution in distilled water) to prepare a slurry, which was then dried at ~100 °C overnight [30]. The dried powder was pelletized (5 mm, pressed under 6 kPa for 15 min) and calcined at 750 or 1000 °C for 1 h under O_2_ atmosphere (atm) (Carbolite Tube Furnace, MTF 12/38/400). Similarly, the as-synthesized samples were pressed into pellets (5 mm diameter, 6 kPa pressure for 15 min) and then gold-coated. Their ionic conductivity was then measured using impedance spectroscopy, and no calcination/heat treatment was performed on these pellets.

XRD was measured by an X-Ray diffractometer (Malvern Empyrean diffractometer PANalytical with Cu Kα radiation; Malvern; UK). Transmission Electron Microscopy (TEM; Jeol JEM-2200FS; Tokyo, Japan) and Scanning Electron Microscopy (SEM; Philips Xl20; Terneuzen, The Netherlands) were used to determine the particles’ morphology, size, and shape. XPS spectra were recorded using a VersaProbe II (ULVAC-PHI; Chigasaki, Japan) equipped with Al K_α_ radiation. Simultaneous thermal analysis (STA) consisting of Thermo Gravimetric Analyses (TGA) and Differential Scanning Calorimetry (DSC) was carried out with a Netsch STA 449 F1 Jupiter (Selb, Germany) under synthetic air with a heating rate of 10 K/min from room temperature up to 1050 °C and combined with gas analysis during sample heating by Quadrupole Mass Spectrometry (QMS 403 D, NETZSCH-Gerätebau GmbH, Selb, Germany). For impedance measurements, a thin gold film was sputtered on both sides of the calcined pellets (Figure 1), and the ionic conductivity of the pellets was measured at room temperature by an impedance analyzer, Solartron 1255, over a 1 Hz to 1 MHz frequency range. The XRD data were fitted with GSAS-II software (Argonne National Laboratory [31]) and MAUD software (version 2.999). The impedance spectra were fitted with Relaxis 3-Impedace Spectrum Analysis software (RHD instruments, Darmstadt, Germany; version 3.0.20.19). The XPS data were fitted with CASA-XPS software (CasaXPS 2.3.25), and the C-C1s signal was used as an internal standard.

## 3. Results

Nanoparticle formation during spray-flame synthesis can lead to the formation of a mixture of two materials morphologies composed of nanoparticles of up to a few tens nm in diameter with a small and narrow particle-size distribution—resulting from a gas-to-particle process. On the other hand, sub-micron-sized particles with diameters of hundreds of nanometers result from a droplet-to-particle conversion [32]. Here, the formation of large-scale hollow and porous particles—which, for some applications, could be of high value—is not desired. In the gas-to-particle process, the precursors completely evaporate into the gas phase and then nucleate from the gas phase. In the droplet-to-particle process, spherical (usually polycrystalline) particles form when the solvent evaporates from the spray droplets. This evaporation causes the dissolved metal oxide precursors to precipitate. The size of these particles depends on the droplet size and the concentration of the precursors within the droplets. Although hydrolysis and precipitation of TiO_2_ in the droplet cannot be completely ruled out, we assume, based on our own experience, that this plays only a minor role here [33].

**Figure 2:**
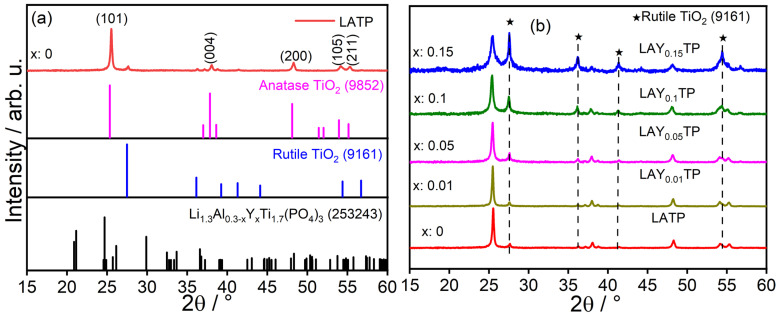
(**a**) XRD of as-synthesized undoped LATP sample (orange graph) and the reference diffraction patterns of anatase, rutile, and LATP; (**b**) XRD comparison of varying Y-doped samples.

As in our previous studies on the production of LLZO and LYZP [24,34], it was not possible to obtain the desired LA(Y)TP phase directly from the spray flame synthesis. We suspect that the main reason is that TiO_2_ easily nucleates from the gas phase. At the same time, Li, Al, and phosphate are all known as glass formers, which later nucleate separately and are, therefore, not directly incorporated into the crystalline structure of the nanoparticles. As a result—as can be seen from the XRD investigations—only titanium dioxide is found as a crystalline phase (as shown in Figure 2a,b).

**Figure 3:**
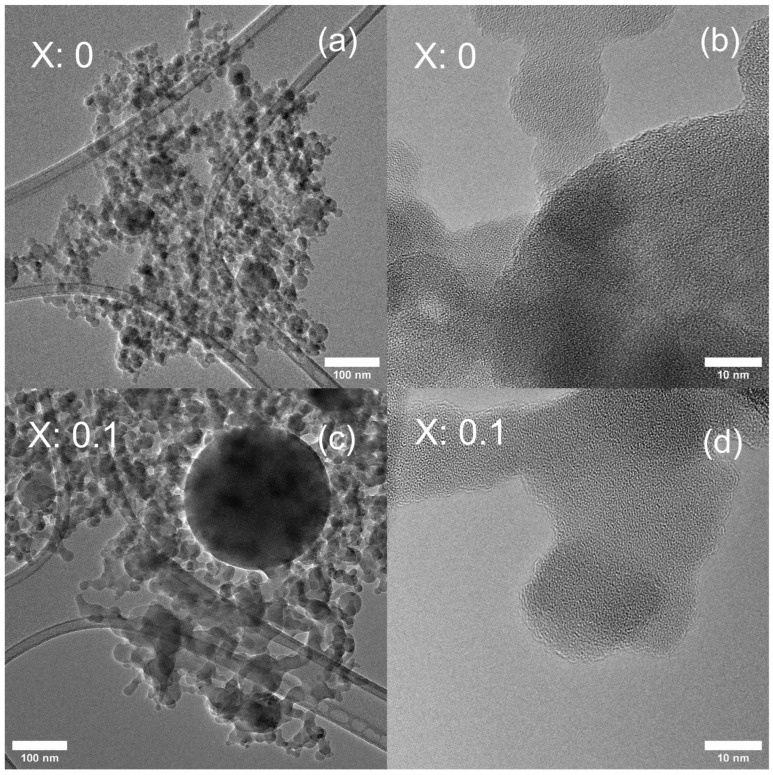
(**a**,**c**) show TEM images of undoped and Y-doped (x: 0.1) samples, respectively; and (**b**,**d**) HRTEM of undoped and Y-doped (x: 0.1) samples, respectively.

The undoped as-synthesized sample LATP (Figure 2a) has tetragonal anatase TiO_2_ [A-TO; ICSD: 9852; space group: I41/amdZ (141)] as a primary phase, and the peaks at 2θ = 25.4, 38.02, 48.2, 54.1, and 55.2° can be assigned to this phase. However, the as-synthesized sample shows traces of rutile TiO_2_ [R-TO; ICSD: 9161; space group: P42/mm (136)] as a secondary phase with peaks at 2θ = 27.6, 37, 41.3, and 54.4°. The XRD results of materials with Y doping are shown in Figure 2b. In all the as-synthesized samples, the primary and impurity phases remain the A-TO and R-TO, respectively. Gribb et al. have demonstrated that the anatase phase is more stable for nanoparticles than the rutile phase [35], while for micrometer-sized samples, the rutile phase is more favored. Da Silva et al. reported similar observations; A-TO’s phase transition crossover to R-TO was ~17 nm [36]. However, Figure 2b shows a significant increment in the R-TO phase with increasing Y doping. It is well-documented that metallic dopants can influence the transition between the rutile and anatase phases of TiO_2_. According to Hanaor et al., small ionic radii (dopants) cations with low (<4) oxidation state promote further A-TO transformation to R-TO [37]. Loan et al. have shown that the rutile phase can become the dominant phase at room temperature (RT) [38] with increasing iron doping and accredited this observation to oxygen vacancies and interstitials supporting Ti^4+^ reduction to Ti^3+^. Furthermore, Da Silva et al. have shown that the surface energy of Nb-doped TiO_2_ decreases with increasing Nb doping concentration, and the phase transformation of 2 mol% Nb_2_O_5_-doped TiO_2_ increases to ~30 nm [36]. Moreover, an oxidizing atmosphere during the SFS also promotes A-TO rather than R-TO, as a reducing/inert atmosphere creates oxygen vacancies, promoting R-TO [39]. To conclude, it is evident from the literature that synthesis conditions, dopant, and the nature of the dopant, etc., can influence the A-TO to R-TO transformation. Even though the SFS’s oxidizing reaction condition and the small nanoparticles’ size (Figure 3) promote the A-TO formation, Y^3+^ doping influences the phase shift to R-TO, resulting in an increasing amount of R-TO in as-synthesized samples with increasing concentration of Y^3+^ (Figure 2a). The results of the Rietveld refinement of the XRD measurements are shown in Table S1 (in Supporting Information). Two different particle sizes of the A-TO phase were chosen to fit the peak broadening.

In addition to primary particles with a size of around 20 nm, whose formation can be explained by the gas-to-particle route, the TEM images shown in Figure 3a and c indicate a small number of larger spherical particles on the sub-micrometer scale, whose occurrence can be explained by the droplet-to-particle route. However, no clear trend of particle size was observed with Y^3+^ doping. For example, the yttrium-free LATP sample has 7.8 wt% of the R-TO phase and increases to 30.4 wt% in the case of the LAY_0.15_TP sample. The Y doping does not affect the particles’ overall size distribution, as summarized in Figure S1 (in the Supporting Information). Figure 3b,d show magnified areas (ten-fold magnification) of Figure 3a,c, and the amorphous phase present in the samples can be easily observed. Figure S2 shows a gradual zoomed-in TEM image of LAY_0.05_TP, indicating in Figure S2c an amorphous phase surrounding a crystalline nanoparticle. This nanoscale mixing of amorphous phases (Li, Y, Al, and phosphate species) and crystalline (A-TO and R-TO) is an essential rationalism for obtaining the LATP phase after a short calcination step (shown later).

**Figure 4:**
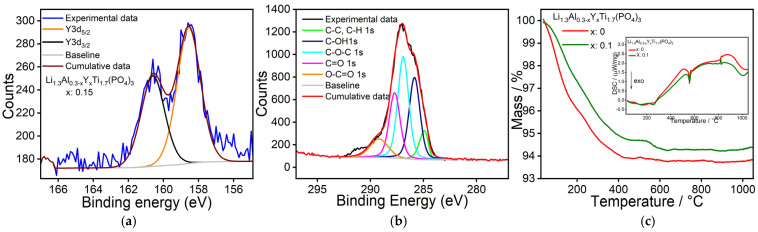
(**a**,**b**) XPS of Y and C elements of as−synthesized LAY_0.15_TP sample, respectively. (**c**) TGA and DSC (inset plot) of LATP and LAY_0.1_TP sample.

XPS experiments were performed to understand further the surface of the as-synthesized samples (Figure 4a,b and Figure S3). Although we only observed the TiO_2_ peak in XRD (Figure 2), the XPS confirms the presence of all elements used. Moreover, XPS analysis also confirms the presence of carbonaceous species (C–O; C=O, etc.) on the surface of the as-synthesized samples (Figure 4b). Figure 4a shows the Y 3d spectra of the LAY_0.15_TP sample. The fitted peak at ~158.5 eV can be assigned to Y (3d_5/2_), and at the separation of ~2.05 eV at 160.6 eV can be assigned to Y^3+^ (3d_3/2_) peaks. These peaks correspond to the Yttrium oxide peak [40]. Similarly, the Ti 2p peak (Figure S3d) could be deconvoluted with two Ti (IV) and Ti (III) peaks, corresponding to 2p_3/2_ and 2p_1/2_ counterparts [41], and agree with well-documented TiO_2_ XPS samples. The Li signal (Figure S3) shows two distinct areas, at ~57 eV, attributed to lithium-carbonate species, and at higher binding energy, ~58.4 eV, as lithium phosphate species [42,43]. This also provides first-hand proof of Li presence on the surface of the as-synthesized LATP samples. The P peak similarly has two distinct (2p_3/2_) peaks at ~133.6 eV and ~134.8 eV, which could be deconvoluted to Li and Ti phosphates [44].

TGA experiments (Figure 4c) were performed on as-synthesized samples to identify and quantify the mass of adsorbed species and to understand the effect of the heat treatment on the doped and undoped samples. Only two samples (Figure 4c) are shown here, as almost all the doped samples show similar results. A gradual mass decrease to ~400 °C was observed and can be attributed to the decomposition and oxidation of physisorbed carbonaceous and water species from the surface (corresponding QMS signals of CO_2_ and H_2_O are shown in Figure S4). To remove unwanted surface adsorbed species but to avoid undesirable reactions, annealing at 350 °C (for 1 h, under air) was chosen (Figure 1) before further processing. This step removes adsorbed impurities, which can hamper the solid-state reaction step and later affect the ionic conductivity.

Choosing the right annealing temperature and duration is always a balance between different factors. On one hand, achieving good ionic conductivity with minimal and clean grain boundaries is desirable, and requires long sintering times and results in the formation of large crystallites. On the other hand, long sintering times at high temperatures always lead to the unwanted formation of foreign phases (preferably at grain boundaries) with poor ionic conductivity blocking Li conduction [45], which must be avoided. Finally, processing time and costs are essential to ensure an economical process.

At higher temperature, the DSC graph (inset of Figure 4c) shows a sharp exothermic peak at ~580 °C together with a minor release of CO_2,_ indicating the formation of LA(Y)TP (Figure S4b), which is in accordance with Monaca et al., who reported LATP phase formation from TiO_2_, Li_2_CO_3_, Al_2_O_3_, and (NH_4_)_2_HPO_4_] below 700 °C [46]. Thus, it can be concluded that the as-synthesized samples, which show an intimate mixing of nanocrystalline TiO_2_ with an amorphous material consisting of lithium, aluminum, yttrium, and phosphate (Figure S2c), undergo a classical solid-state reaction during calcination, which can be expected to occur at a relatively low temperature [22]. Respectively, the first calcination temperature for our annealing process (Figure 1) was selected at 750 °C. Since the DSC graph shows a second, minor signal around 827 °C, which most probably originates from the melting of Li_3_PO_4_ (melting point 837 °C) [21], the calcination temperature for a second series of annealing tests was set at 1000 °C.

## 4. Discussion

**Figure 5:**
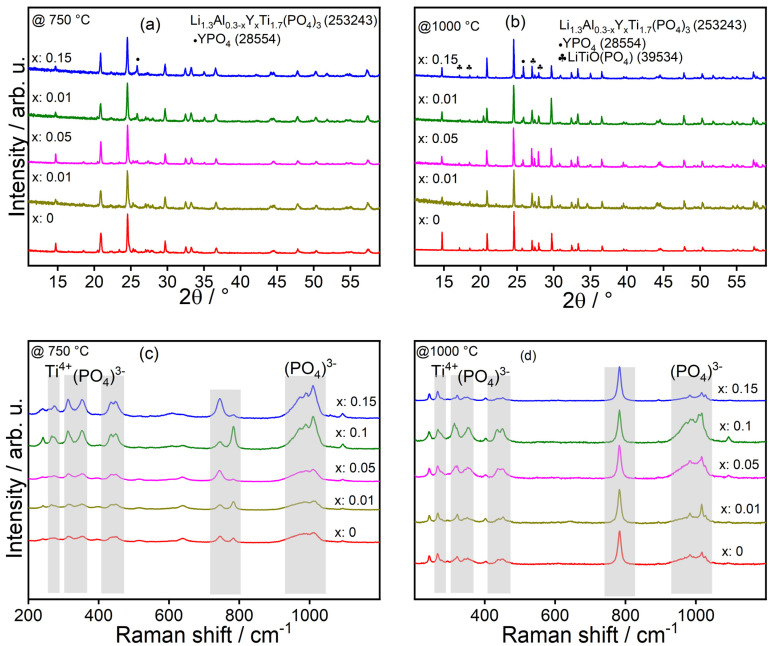
(**a**,**b**) show the comparison XRD of calcined samples at 750 and 1000 °C, respectively. (**c**,**d**) show the comparison of Raman spectra of calcined samples.

The annealing at 750 and 1000 °C was performed for 1 h under O_2_ atm, and the respective samples’ XRD data are shown in Figure 5a,b. The 2θ peaks at 14.8, 21.10, 24.7, 25.7, 26.2, 29.9, 32.4, 33.7, 36.7, etc., can be attributed to the LATP phase irrespective of the calcination temperature. Depending on Y content, a clear peak shift to lower 2θ could be observed for all samples as indicated for 2θ = 24.7° (Figure S5a,b), suggesting a successful doping of the LATP phase. However, peaks at 2θ = 27.1 and 28.01° could be assigned to LiTiOPO_4_ (LTOP; ICSD: 39534; space group: P n m a (62); orthorhombic crystal structure) as an impurity phase and are much more prominent in the case of the samples calcined at 1000 °C. Its phase fraction is, in all cases, much higher with a change to the calcination temperature from 750 °C to 1000 °C (Table S2).

Furthermore, with increasing Y in the system, impurities of YPO_4_ [YPO; space group: I 41/a m d Z (141), tetragonal crystal structure] could be identified due to the occurrence of signals at 2θ = 25.9, 34.9°. Rietveld analysis demonstrates that the LAY_0.05_TP sample shows the first appearance of the YPO phase, and it increases with increasing Y concentration, e.g., LAY_0.15_TP sample calcined at 750 °C has 9.1 wt% of YPO (Figure S6 and Table S2 in the Supporting document).

As the peak shift suggests, a steady increase in unit cell volume of NASICON structure can be expected with increasing Y doping [47] as the relatively larger Y^3+^ (0.93 Å) displaces the smaller Al^3+^ (0.53 Å) and Ti^4+^ (0.60 Å), which increases the unit cell volume [47]. We do not expect competition between Al^3+^ and Y^3+^, but rather substitutional doping replacing Ti^4+^, as the Ti concentration is more than five times higher than that of Al [48]. Moreover, doping with yttrium also encourages the formation of a YPO-segregated phase, thus explaining the formation of the YPO phase in our sample [49].

Similar to the XRD results, analogous results are also observed from Raman measurements of the calcined samples (Figure 5c,d). The broad peaks from ~860 to 1150 cm^−1^ and ~300 to 500 cm^−1^ are attributed to the stretching and bending of P–O bonds [50]. The peaks between 250 and 300 cm^−1^ are transitional vibrations of Ti^4+^ atoms. However, interestingly, there is a strong peak at ~784 cm^−1^ for samples calcined at 1000 °C (Figure 5d). Moreover, in the same region, there are two peaks of varying intensity with varying Y incorporation for samples annealed at 750 °C (Figure 5c). According to Li et al., the peak at ~784 cm^−1^ can be assigned to the vibration mode of the O–Metal–O bond [51], and we assume that it is related to the O–Ti–O bond of the LiTiOPO_4_ phase.

**Figure 6:**
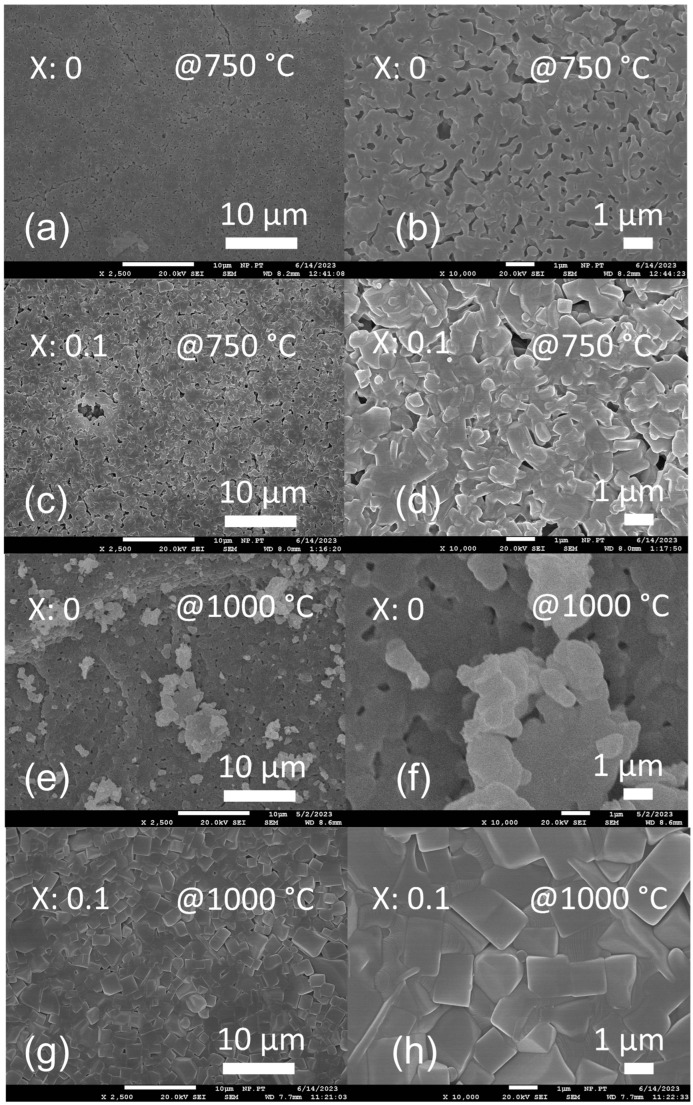
(**a**,**c**) Fare ESEM images of LATP@750, LAY_0.1_TP@750 °C. (**b**,**d**) are zoomed images of (**a**) and (**c**), respectively. (**e**,**g**) are FESEM images of LATP@1000, LAY_0.1_TP@1000 °C. (**f**,**h**) are zoomed images of (**e**) and (**g**), respectively.

To investigate the microstructure of the different materials, FESEM was performed on LATP and LAY_0.1_TP samples annealed at 750 and 1000 °C (Figure 6). Although the sintered materials essentially have very similar microstructures, there are still specific differences. While the crystallites of the undoped LATP are more spherical, those of the LAY_0.1_TP show a much stronger faceting and, especially at 1000 °C, a much more substantial crystallite growth, combined with a lower porosity (Figure 6). The driving force can be the (intermediate) formation of lithium phosphate as well as the formation of LiTiOPO_4_ as a sintering aid (due to their comparatively low melting temperatures, see Table S2 [52,53]. Moreover, the formation of the high-temperature stable YPO_4_ might have an additional impact in the case of LAY_0.1_TP. In summary, it can be stated that doping with yttrium leads to larger crystallites.

It is well known that impurity phases, phase segregation, number of grain boundaries, and space charges significantly affect the ionic conductivity of ceramics, specifically inter-grain and interfacial conductivity [54]. We have already shown in Table S2 that Y doping and calcination temperature affect the segregation of YPO and LTOP. Especially for 10 and 15% doping and annealing at 1000 °C, it is evident that an increasing amount of the YPO phase is formed. However, segregation of YPO and impurity phase presence can also backfire if it reaches a threshold. Excess YPO formation removes the dopant Y and, in addition, results in an ionic inhibitor layer. Thus, the microstructure and phase content maintain a crucial balance regarding doping concentration and sintering temperature.

**Figure 7:**
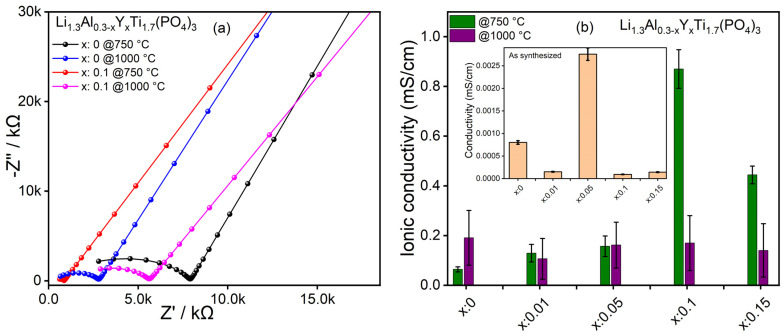
(**a**) Impedance spectra of calcined LATP and LAY_0.1_TP samples. (**b**) comparison of the specific ionic conductivity between calcined samples and pelletized as-synthesized samples (inset plot). The specific conductivities were calculated from the impedance values, considering the slightly different sample geometries.

In our case, doping LATP with yttrium acts as a sintering aid, significantly increasing the sintering activity, as seen from the comparison of Figure 7a,b. This means that LAY_0.1_TP already has significantly fewer grain boundaries than the undoped LATP sintered at 750 °C but, at the same time, the disturbing influences of foreign phases are still low. Similar behavior is also shown by sample LAY_0.15_TP, whose conductivity is also higher than that of the other samples. However, after sintering at 1000 °C, the proportion of foreign phases increases significantly in all cases (Table S2), which cannot be compensated by improved conductivity due to the reduction in grain boundaries. Accordingly, the combination of “sintering aid” and sintering temperature represents a suitable compromise for LAY_0.1_TP sintered at 750 °C. Similarly, LAY_0.15_TP@750 °C still shows higher ionic conductivity than its undoped counterpart (Figure 7) [45,55]. Here, we report the highest ionic conductivity of 0.84 mS/cm for LAY_0.1_TP@750 °C, which is remarkably higher than ~0.1 mS/cm for undoped LATP@750 °C samples Figure 7b.

Our approach in this manuscript is to use a scalable, continuous manufacturing process to produce nanoscale materials that can be processed into functional ceramics with good ionic conductivity at comparatively low sintering temperatures. It is known from the literature that materials with comparable properties can be produced using conventional methods such as solid-state reactions and sol-gel processes. Zhao et al. have prepared a series of Y-doped (Li_1.3_Al_0.3−x_Y_x_Ti_1.7_(PO_4_)_3_ (x = 0, 0.025, 0.05, 0.075, 0.15)) LATP by modified solid-state reaction [45]. The authors observed the highest ionic conductivity of 0.78 mS/cm (x = 0.075), which is very similar to our result of ~ 0.84 mS/cm (LAY_0.1_TP). However, the significance of SFS over solid-state reaction is that the total calcination time for our case is only 1 h at 750 °C. In the case of Zhao et al., the minimum time for calcination was 8 h, with an extra 4 h of ball milling in between [45]. Hence, the SFS presents a comparable ionic conductivity with less time and fewer steps and, eventually, with a low-cost requirement.

## 5. Conclusions

The processing of suitable precursor solutions to produce yttrium-doped LATP leads in the first step to the formation of crystalline TiO_2_ surrounded by an amorphous layer consisting of lithium, aluminum, yttrium, and phosphate. Simultaneous thermal analysis under synthetic air showed that temperatures around 700 °C should be sufficient to produce the desired material, confirmed by annealing at 750 °C for one hour to obtain the LATP NASICON phase. Sintering for one hour at 1000 °C yielded materials that showed significant crystallite growth compared to annealing at 750 °C. With Y doping, a subsequent increase in rutile TiO_2_ phase content was observed in as-synthesized samples. YPO_4_ phase separation was observed at higher Y-doped samples for both the calcined samples. However, the LiTiOPO_4_ phase was also present irrespective of Y doping for 1000 °C calcined samples, serving as a suitable sintering aid due to its comparatively low melting temperature of 1085 °C.

Our investigations suggest a complex relationship between dopant content, sintering temperature, and the formation of unwanted impurity phases, in this case YPO_4_, concerning improving ionic conductivity. Ultimately, Y-doped LAY_0.1_TP with substitutional doping of 10 % yttrium demonstrated high ionic conductivity of 0.84 mS/cm at room temperature, significantly higher than the undoped sample (~0.1 mS/cm), indicating a notable improvement in material performance. Compared to previous studies (e.g., [50,56], Table S3), our work demonstrates enhanced ionic conductivity, relatively lower sintering temperature, and reduced impurity phase formation, making it a promising candidate for solid electrolytes in energy storage applications.

We conclude that incorporating yttrium can significantly enhance the ionic conductivity of LATP by altering the phase composition and microstructure. This study introduces a novel approach to doping and sintering conditions to optimize the material properties. Furthermore, this report establishes spray-flame synthesis as an alternative synthesis technique for producing ceramic solid electrolytes with less time, fewer steps, and low-cost requirements.

Future Work: Future research should focus on further optimizing doping levels and sintering conditions to enhance material performance and exploring the scalability of the spray-flame synthesis method for industrial applications.

## Figures and Tables

**Table 1:** Spray-flame reactor synthesis parameters.

Operating Parameters						
Dispersion Gas CH_4_ (slm)	Dispersion Gas O_2_ (slm)	Pilot Flame CH_4_ (slm)	Pilot Flame O_2_ (slm)	Quench Gas Air (slm)	Coaxial Sheath Air (slm)	Reactor Pressure (mbar)
1	9	2	16	240	140	800–820

**Table 2:** Scheme of precursors and their corresponding terminology.

Nomenclature		Precursors					Solvents
		LN	YN	AN	TTIP	TBP	Propanol + Propionic Acid (1:1)
Li_1.3_Al_0.3_Ti_1.7_(PO_4_)_3_	LATP	✓ (25% excess)	×	✓	✓	✓	✓
Li_1.3_Al_0.29_Y_0.01_Ti_1.7_(PO_4_)_3_	LAY_0.01_TP		✓	✓	✓	✓	
Li_1.3_Al_0.25_Y_0.05_Ti_1.7_(PO_4_)_3_	LAY_0.05_TP		✓	✓	✓	✓	
Li_1.3_Al_0.2_Y_0.1_Ti_1.7_(PO_4_)_3_	LAY_0.1_TP		✓	✓	✓	✓	
Li_1.3_Al_0.15_Y_0.15_Ti_1.7_(PO_4_)_3_	LAY_0.15_TP		✓	✓	✓	✓	

## Data Availability

Data can be made available upon request.

## Supplementary Materials

The following supporting information can be downloaded at: https://www.mdpi.com/article/10.3390/nano15010042/s1, Table S1. Rietveld analysis of XRD of as-synthesized samples; Figure S1. Particle-size distribution of (a) LATP (average particle size: 15.6 nm) and (b) LAY_0.1_TP (average particle size: 17.4 nm) as-synthesized samples; Figure S2. TEM (a) and HRTEM (b) image of LAY_0.05_TP nanoparticles. The (b) image shows the zoomed image of (a). The marked big (~300 nm) spherical particle has multiple crystalline small particles attached to its surface. One of these nanoparticles’ crystalline fringes (A-TO or R-TO) are marked in (c); Figure S3. (a), (b), (c), (d), and (e) show the XPS spectra of Al 2p, O 1s, Li 1s, Ti 2p, and P 2p, respectively, of the LAYTP sample; Figure S4 TGA-QMS signal of (a) H_2_O, (b) CO_2_ released during TGA experiment of doped and undoped samples; Figure S5. (a) & (b) show the comparison XRD (in between 2θ: 23 to 26) of calcined samples at 750 and 1000 °C, respectively; Table S2. Phase fraction of calcined samples; Figure S6. Rietveld refinement of LAY0.15TP sample calcined at 750 (a) and 1000 °C (b), respectively; Table S3. Literature comparison of doped LATP solid-electrolyte. References [57,58,59,60,61] are in the supplementary materials.
