# Supplementary material for: Spray-Flame Synthesis (SFS) and Characterization of Li1.3Al0.3−xYxTi1.7(PO4)3 [LA(Y)TP] Solid Electrolytes
Source: Nanomaterials (Basel). 2024 Dec 29;15(1):42. doi: 10.3390/nano15010042 (PMC11723016; doi:10.3390/nano15010042)
Supplement: Supplementary file 1 [file nanomaterials-15-00042-s001.zip › nanomaterials-3363464-supplementary.pdf]

nanomaterials

Supplementary Information

Spray-Flame Synthesis (SFS) and Characterization of $Li_{1.3}Al_{0.3-x}Y_xTi_{1.7}(PO_4)_3$ [LA(Y)TP] Solid Electrolytes

Md Yusuf Ali [1], Hans Orthner [1] and Hartmut Wiggers [1,2,*]

[1] Institute for Energy and Materials Processes—Reactive Fluids, University of Duisburg-Essen, 47057 Duisburg, Germany; yusuf.ali@uni-due.de (M.Y.A.); hans.orthner@uni-due.de (H.O.)
[2] Center for Nanointegration Duisburg-Essen (CENIDE), 47057 Duisburg, Germany
* Correspondence: hartmut.wiggers@uni-due.de

Table S1. Rietveld analysis of XRD of as-synthesized samples.

Samples	Anatase1 (9852) (wt%)	Size (nm)	Anatase2 (9852) (wt%)	Size (nm)	Rutile (9161) (wt%)	Size (nm)
LATP	53.2	18.4	40.9	89.5	5.8	68.8
$LAY_{0.01}TP$	25.6	20.6	64.3	95	10.2	60
$LAY_{0.05}TP$	23.2	32.7	60.7	52.5	16.03	49.5
$LAY_{0.1}TP$	45.3	35.6	30.7	56.2	23.9	56.1
$LAY_{0.15}TP$	43	7.6	26.6	26.6	30.4	46.1

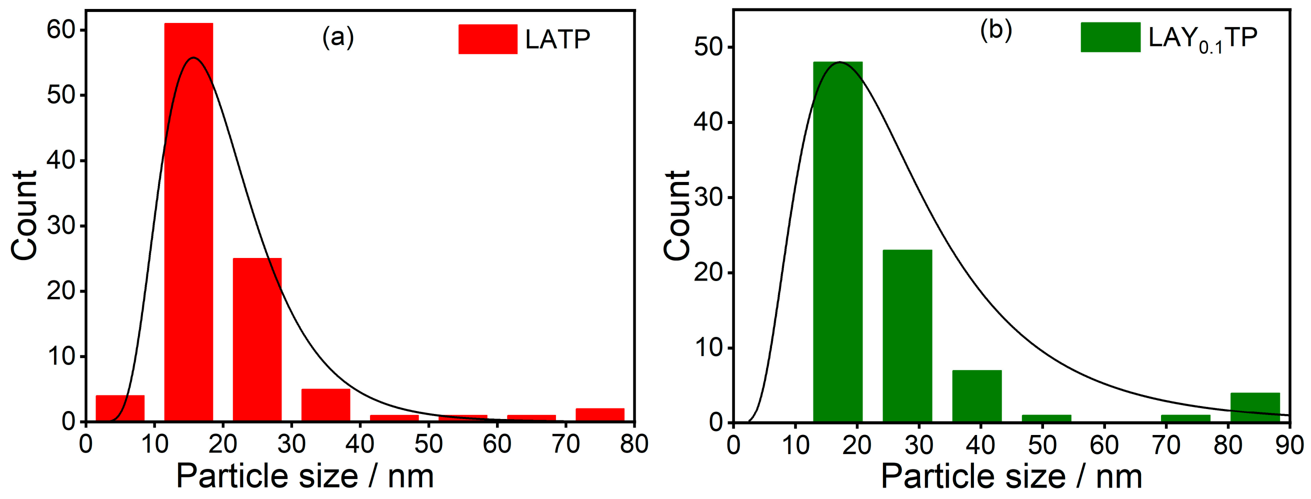

Figure S1. Particle-size distribution of (a) LATP (average particle size: 15.6 nm) and (b) $LAY_{0.1}TP$ (average particle size: 17.4 nm) as-synthesized samples.

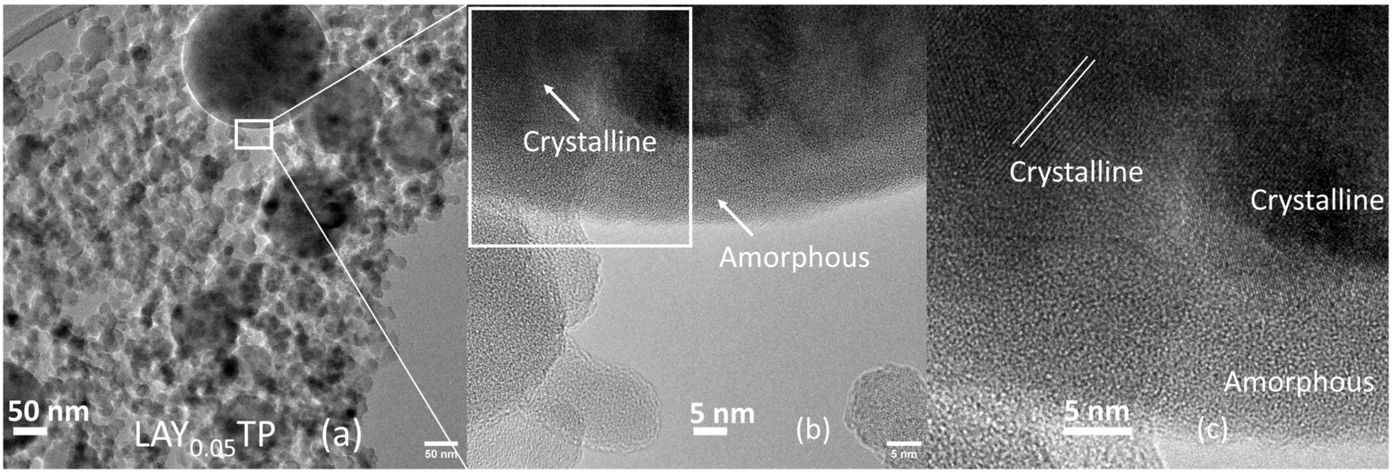

Figure S2. TEM (a) and HRTEM (b) image of $LAY_{0.05}TP$ nanoparticles. The (b) image shows the zoomed image of (a). The marked big (~ 300 nm) spherical particle has multiple crystalline small particles attached to its surface. One of these nanoparticles' crystalline fringes (A-TO or R-TO) are marked in (c).

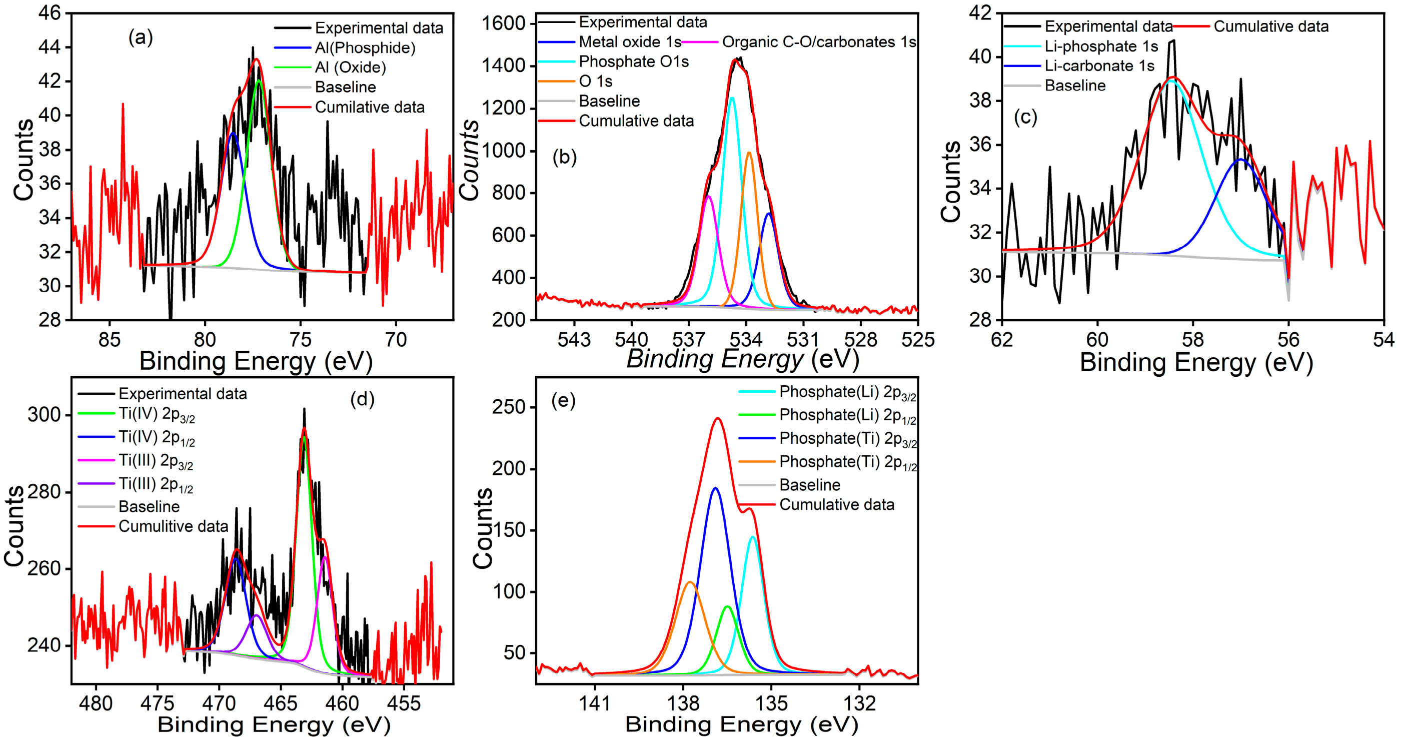

Figure S3. (a), (b), (c), (d), and (e) show the XPS spectra of Al 2p, O 1s, Li 1s, Ti 2p, and P 2p, respectively, of the LAYTP sample.

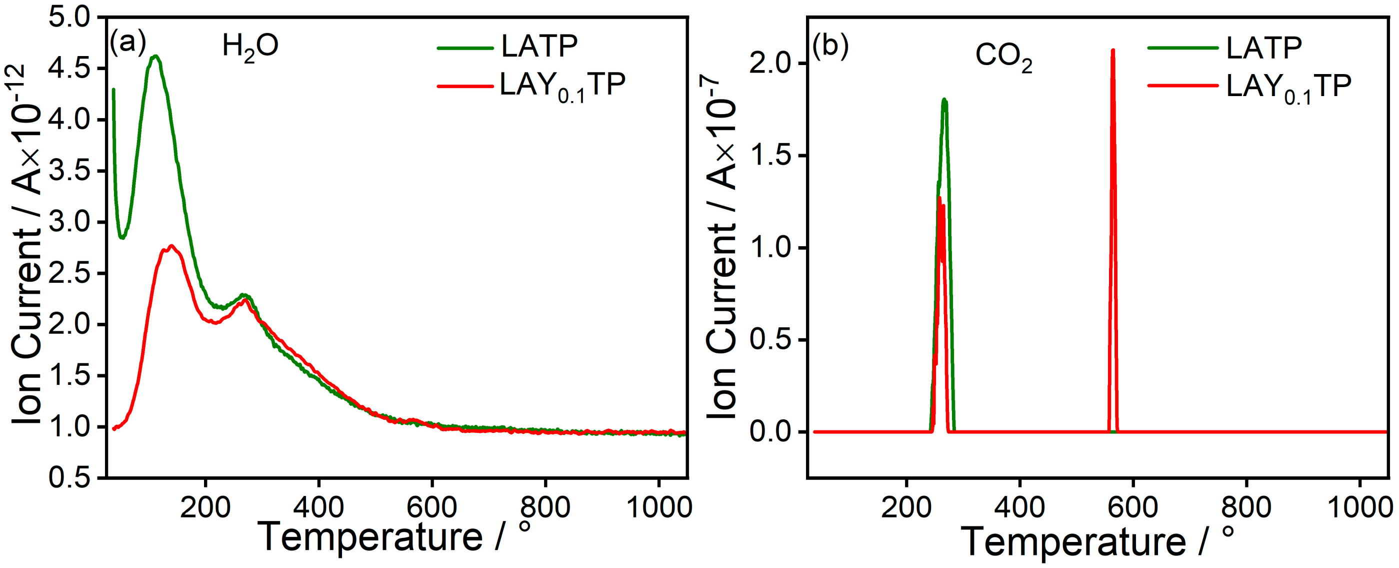

Figure S4. TGA-QMS signal of (a) H_2O, (b) CO_2 released during TGA experiment of doped and undoped samples.

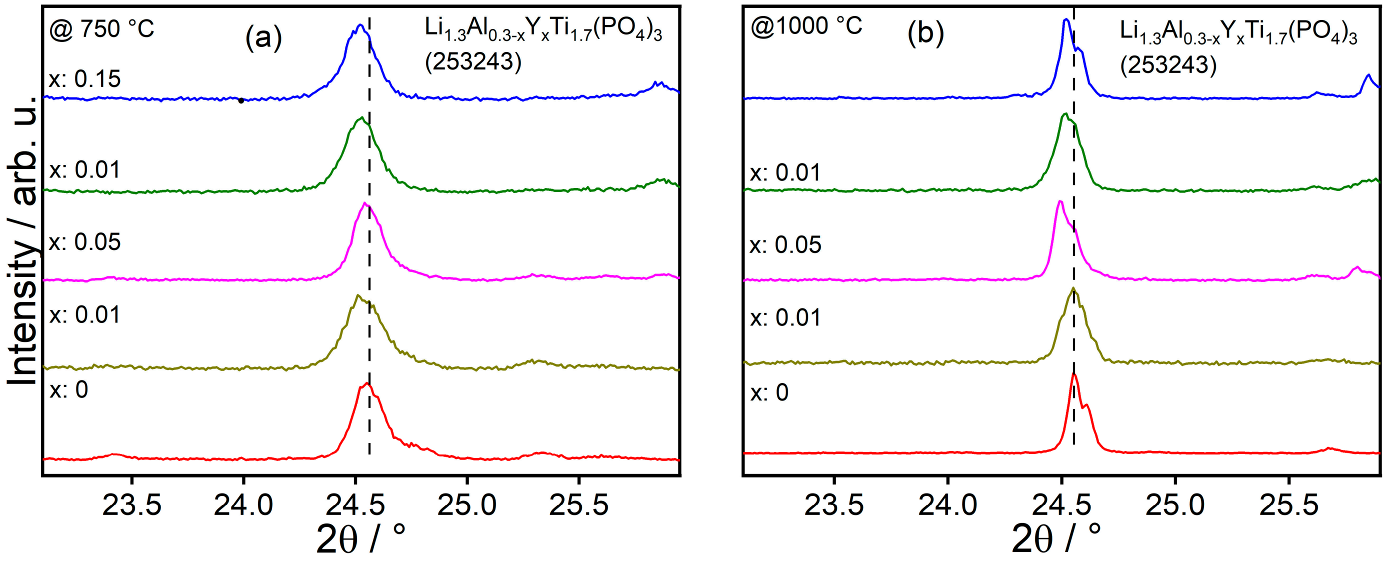

Figure S5. (a) & (b) shows the comparison XRD (in between 2θ: 23 to 26) of calcined samples at 750 and 1000 °C, respectively.

Table S2. Phase fraction of calcined samples.

**Sample**	**Phase fraction (wt %)**			
	**Calcination Temperature (°C)**	**LATP (253243)**	**$LiTiOPO_4$ (39534)**	**YPO_4 (28554)**
**Melting Temp.**			1085 °C [1]	1995 °C [2]
LATP	750	89.8	10.2	--
	1000	71.2	27.3	--
$LAY_{0.01}TP$	750	83.9	16.1	--
	1000	68.2	31.8	--
$LAY_{0.05}TP$	750	89.4	8.6	2
	1000	57.7	38.6	3.7
$LAY_{0.1}TP$	750	83.8	10.3	5.9
	1000	71.5	22.2	6.3
$LAY_{0.15}TP$	750	87.1	3.7	9.1
	1000	69.1	22.5	8.4

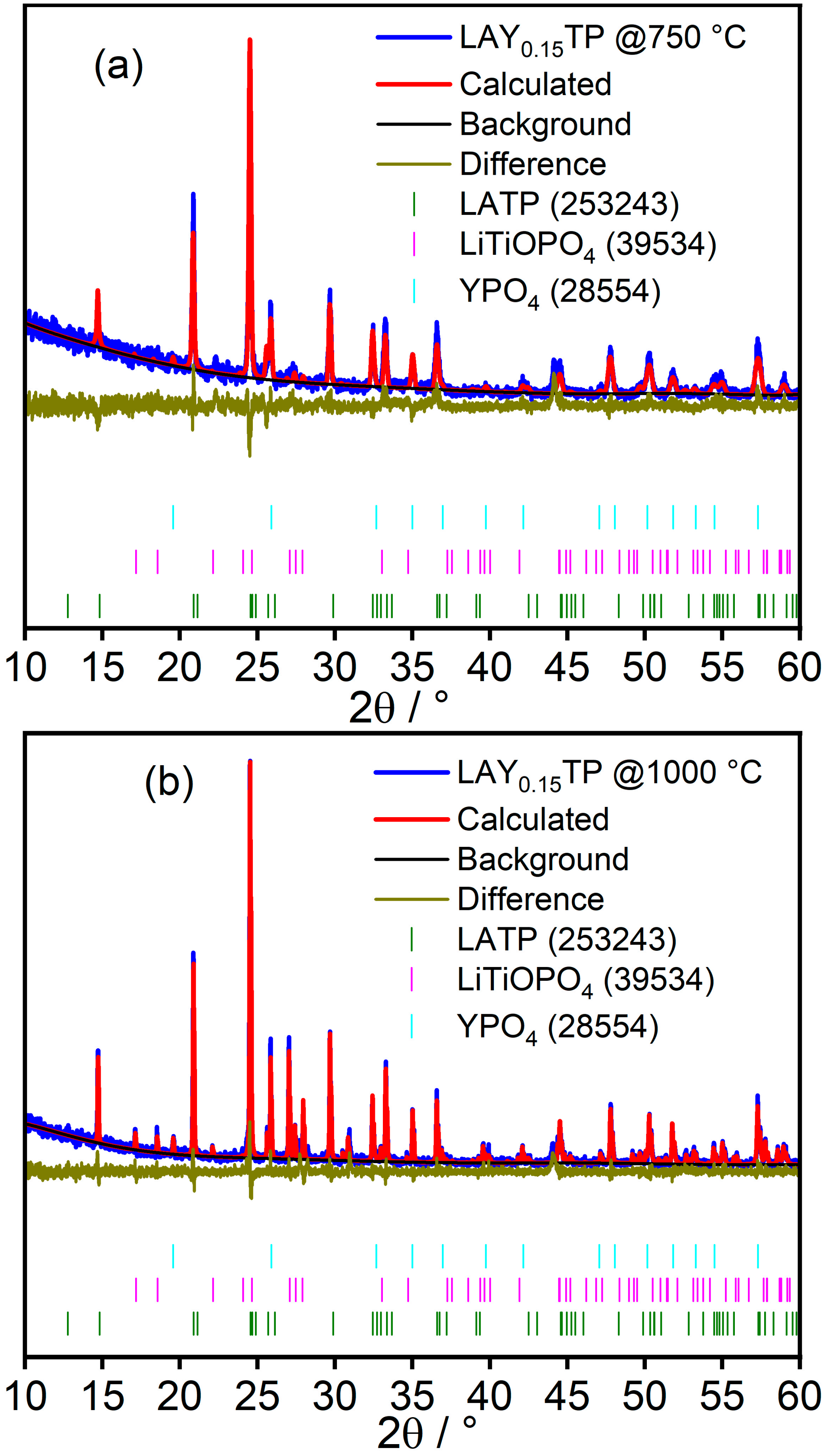
(a)
LAY0.15TP @750 °C
Calculated
Background
Difference
LATP (253243)
LiTiOPO4 (39534)
YPO4 (28554)
10 15 20 25 30 35 40 45 50 55 60
2θ / °
(b)
LAY0.15TP @1000 °C
Calculated
Background
Difference
LATP (253243)
LiTiOPO4 (39534)
YPO4 (28554)
10 15 20 25 30 35 40 45 50 55 60
2θ / °

Figure S6. Rietveld refinement of LAY0.15TP sample calcined at 750 (a) and 1000 °C (b), respectively.

Table S3. Literature comparison of doped LATP solid-electrolyte.

Sample	temperature (°C)	Time (h)	Single step	Ionic conductivity (mS/cm)	Year	Ref.
W-doped LATP	850	4	×	1.186	2024	[3]
Si-doped LATP	900	6	×	0.00169	2022	[4]
LATP	1050 (SPS)	0.083	×	1.0	2020	[5]
B doped LATP	900	5	×	0.24	2024	[6]
Ga doped LATP	1100	5	×	~1	2016	[7]
Ga doped LATP	230Í	48	×	0.23	2019	[8]
Zr doped LATP	900	6	×	0.023	2020	[9]
B doped LATP	1200	1	yes	0.12	2020	[10]
Sn doped LATP	900	6	×	0.47	2022	[11]
Y doped LATP	750	1	×	0.84	this work	

SPS: Spark plasma synthesis; Í: Autoclave hydrothermal reaction

References

1. Robertson, A.; Fletcher, J.G.; Skakle, J.M.S.; West, A.R. Synthesis of $LiTiPO_5$ and $LiTiAsO_5$ with the α-Fe_2PO_5 Structure. *Journal of Solid State Chemistry* **1994**, *109*, 53-59, doi:https://doi.org/10.1006/jssc.1994.1070.
2. Hikichi, Y.; Nomura, T. Melting Temperatures of Monazite and Xenotime. *Journal of the American Ceramic Society* **1987**, *70*, C‐252-C‐253, doi:https://doi.org/10.1111/j.1151-2916.1987.tb04890.x.
3. Guo, Y.; Zhao, E.; Li, J. Superior ionic conductivity of W-doped NASICON-type $Li_{1.3}Al_{0.3}Ti_{1.7}(PO_4)_3$ solid electrolyte. *Journal of the European Ceramic Society* **2024**, *44*, 7081-7091, doi:https://doi.org/10.1016/j.jeurceramsoc.2024.05.005.
4. Zhu, J.; Xiang, Y.; Zhao, J.; Wang, H.; Li, Y.; Zheng, B.; He, H.; Zhang, Z.; Huang, J.; Yang, Y. Insights into the local structure, microstructure and ionic conductivity of silicon doped NASICON-type solid electrolyte $Li_{1.3}Al_{0.3}Ti_{1.7}P_3O_{12}$. *Energy Storage Materials* **2022**, *44*, 190-196, doi:https://doi.org/10.1016/j.ensm.2021.10.003.
5. Waetzig, K.; Rost, A.; Heubner, C.; Coeler, M.; Nikolowski, K.; Wolter, M.; Schilm, J. Synthesis and sintering of $Li_{1.3}Al_{0.3}Ti_{1.7}(PO_4)_3$ (LATP) electrolyte for ceramics with improved Li^+ conductivity. *Journal of Alloys and Compounds* **2020**, *818*, 153237, doi:https://doi.org/10.1016/j.jallcom.2019.153237.
6. Öksüzoğlu, F.; Ateş, Ş.; Özkendir, O.M.; Çelik, G.; Eker, Y.R.; Baveghar, H.; Basyooni-M. Kabatas, M.A. The Impact of Boron Compounds on the Structure and Ionic Conductivity of LATP Solid Electrolytes. *Materials* **2024**, *17*, doi:10.3390/ma17153846.
7. Kothari, D.H.; Kanchan, D.K. Effect of doping of trivalent cations Ga^{3+}, Sc^{3+}, Y^{3+} in $Li_{1.3}Al_{0.3}Ti_{1.7}(PO_4)_3$ (LATP) system on Li^+ ion conductivity. *Physica B: Condensed Matter* **2016**, *501*, 90-94, doi:https://doi.org/10.1016/j.physb.2016.08.020.
8. Liang, Y.; Peng, C.; Kamiike, Y.; Kuroda, K.; Okido, M. Gallium doped NASICON type $LiTi_2(PO_4)_3$ thin-film grown on graphite anode as solid electrolyte for all solid state lithium batteries. *Journal of Alloys and Compounds* **2019**, *775*, 1147-1155, doi:https://doi.org/10.1016/j.jallcom.2018.10.226.
9. Rai, K.; Kundu, S. Fabrication and performances of high lithium-ion conducting solid electrolytes based on NASICON $Li_{1.3}Al_{0.3}Ti_{1.7-x}Zr_x(PO_4)_3$ ($0 \leqslant x \leqslant 0.2$). *Ceramics International* **2020**, *46*, 23695-23705, doi:https://doi.org/10.1016/j.ceramint.2020.06.143.
10. Abdel-Hameed, S.A.M.; Fathi, A.M.; Elwan, R.L.; Margha, F.H. Effect of F^- and B^{3+} ions and heat treatment on the enhancement of electrochemical and electrical properties of nanosized $LiTi_2(PO_4)_3$ glass-ceramic for lithium-ion batteries. *Journal of Alloys and Compounds* **2020**, *832*, 154943, doi:https://doi.org/10.1016/j.jallcom.2020.154943.
11. Xu, A.; Wang, R.; Yao, M.; Cao, J.; Li, M.; Yang, C.; Liu, F.; Ma, J. Electrochemical Properties of an Sn-Doped LATP Ceramic Electrolyte and Its Derived Sandwich-Structured Composite Solid Electrolyte. *Nanomaterials* **2022**, *12*, doi:10.3390/nano12122082.

Disclaimer/Publisher's Note: The statements, opinions and data contained in all publications are solely those of the individual author(s) and contributor(s) and not of MDPI and/or the editor(s). MDPI and/or the editor(s) disclaim responsibility for any injury to people or property resulting from any ideas, methods, instructions or products referred to in the content.
